# The Prevalence and Correlative Factors of Depression Among Chinese Teachers During the COVID-19 Outbreak

**DOI:** 10.3389/fpsyt.2021.644276

**Published:** 2021-06-29

**Authors:** Jiaojiao Zhou, Xiaofei Yuan, Huanhuan Huang, Yaqiong Li, HongYe Yu, Xu Chen, Jia Luo

**Affiliations:** The National Clinical Research Center for Mental Disorders and Beijing Key Laboratory of Mental Disorders, Beijing Anding Hospital and The Advanced Innovation Center for Human Brain Protection, Capital Medical University, Beijing, China

**Keywords:** prevalence, factor, depression, teacher, COVID-19

## Abstract

**Background:** Epidemiological data on outbreak-associated depression of Chinese teachers are not available. This study aimed to investigate the prevalence and correlates of depression among teachers during the coronavirus disease 2019 (COVID-19) outbreak in mainland China.

**Methods:** A large cross-sectional online survey was conducted during the COVID-19 outbreak. Depression was assessed using the Patient Health Questionnaire-9 (PHQ-9). The Connor-Davidson Resilience Scale 25 (CD-RISC 25) and Perceived Stress Scale-10 (PSS-10) were used to measure the mental resilience and stress of participants. The correlative factors of depression were analyzed.

**Results:** In this study, 1,096 teachers were analyzed with a median (range) age of 41 (20–65) years. Of them, 624 (56.9%) suffered from depression (PHQ-9 total score of >4). The multivariate analyses showed that participants with aged ≥41 years (OR = 0.752, 95% CI:0.578–0.979, *p* = 0.034), participating in epidemic prevention and control (OR = 1.413, 95% CI:1.070–1.867, *p* = 0.015), thinking prolonged school closure have bad effect (OR = 1.385, 95% CI:1.017–1.885, *p* = 0.038), sleep duration/day of <6 h (OR = 1.814, 95% CI:1.240–2.655, *p* < 0.001), physical exercise duration/day of <30 min (OR = 1.619, 95% CI:1.247–2.103, *p* < 0.001), spending less time with family (OR = 1.729, 95% CI: 1.063–2.655, *p* = 0.002), being concerned about COVID-19 (OR = 0.609, 95% CI:0.434–0.856, *p* = 0.004), having poor mental resilience (OR = 6.570, 95% CI:3.533–12.22, *p* < 0.001) and higher PSS-10 scores (OR = 9.058, 95% CI:3.817–21.50, *p* < 0.001) were independently associated with depression.

**Conclusion:** During the COVID-19 outbreak, depression was common among teachers. Age, participating in epidemic prevention and control, opinions toward distant teaching and prolonged school closure, sleep duration/day, physical exercise duration, spending time with family, attitude toward COVID-19, mental resilience and stress represented the independent factors for suffering from depression.

## Introduction

Depression, a common mental disorder with severe psychomotor, affective, and cognitive sequelae (1), is a top public health concern for its high prevalence (2), chronicity (3), as well as grave ramifications, which include increased societal costs (4, 5), disability (5), and mortality (5, 6). Teaching is a not only physically but also mentally challenging occupation, and teachers use a lot of energy in daily work in addition to their familial and personal commitments, all of which are continuous source of stress (7, 8). And teaching was proved to be one of the most stressful jobs in a previous study of 26 jobs (9). Occupational stress can reduce the overall physical and mental well-being of teachers and lead to depression (7). Teachers were consistently reported to be at high risk of depression compared to those in other jobs (10, 11). Previous studies indicated that higher levels of depression may lead to teachers under performing at work (12, 13). As frontline professionals who have daily contact with students, teachers play an important role to play in the well-being and mental health of their students (14, 15). Therefore, early prevention, detection and treatment are essential to protect teachers from depressive symptoms and improve their mental health.

At the end of 2019, the corona virus disease 2019 (COVID-19) was believed to have started from Wuhan, Hubei Province, China and has gained great attention nationwide and globally (16, 17). On March 11, 2020, the COVID-19 was declared as a pandemic by the World Health Organization (WHO) (18). As of November, 2020, the COVID-19 outbreak has resulted in more than 50 million confirmed cases and more than 1.2 million deaths globally, which were far beyond the number of those caused by Middle East respiratory syndrome (MERS) and SARS in 2013 and 2003, respectively. Thus, the epidemic has caused considerable challenges worldwide.

The COVID-19 outbreak had a significant negative impact on daily life and mental health among teachers, due to negative life event and heavy occupational stress, resulting in high risk of depressive symptoms, such as sense of uncertainty, boredom, fear, anger, and loneliness associated with quarantine and challenges due to conflict with children, changes in teaching methods, and insufficient outdoor activities. In addition, the COVID-19 outbreak has raised considerable challenges on mental health services among teachers. So, it is the responsibility of all stakeholders to ensure that the mental and physical impact of the outbreak of COVID-19 on teachers is minimal (18).

However, to date, epidemiological data on outbreak-associated depression among Chinese teachers is not available. It is needed to conduct a nationwide survey to determine the prevalence and correlates of depressive symptoms among teachers during the outbreak of COVID-19 in mainland China. The present study aimed to investigate the prevalence and correlates of depression among teachers during the coronavirus disease 2019 (COVID-19) outbreak in mainland China.

## Methods

### Study Design and Participants

This was a national cross-sectional, online survey conducted between April 10 and 17, 2020, based on the collaborative research network of the National Clinical Research Center for Mental Disorders, China. Using a snowball sampling method, data were collected with the WeChat-based Wenjuanxing program (https://www.wjx.cn/). The Wenjuanxing program has been widely used in online survey during the COVID-19 pandemic. The inclusion criteria were as follows: (1) the primary, middle, high school and college teachers aged between 20 and 65 years old and; (2) living in mainland China during the COVID-19 epidemic. Participants who completed the questionnaire in <120 s or were not aged 20–65 years were excluded. All participants provided written informed consent. And this study was approved by the Medical Ethical Committee in Beijing Anding Hospital of the Capital Medical University, China.

### Assessment Instruments and Data Collection

In the present study, a data collection sheet was used to collect socio-demographic and clinical characteristics such as gender, age, marital status, education background, professional title, school type, participating in epidemic prevention and control, being a parent, parent-child relationship, teaching graduating class, participating in distant teaching, opinions toward distant teaching and prolonged school closure, increased working intensity and time, sleep duration/day, physical exercise duration, spending time with family, attitude toward COVID-19, and presence of depression.

The presence of depression was assessed by the self-reported 9 item-Patient Health Questionnaire (PHQ-9), Chinese version (19, 20). The total score ranges from 0 to 27 (1–4: no depression, 5–9: mild depression, 10–14: moderate depression, 15–19: moderately severe depression, and 20–27: severe depression) (19). The test-retest reliability of PHQ-9 ranges from 0.76 to 0.89, and the Cronbach's alpha ranges from 0.79 to 0.89 (21).

The Connor-Davidson Resilience Scale 25 (CD-RISC 25) was developed as a brief self-evaluation instrument to help quantify participant mental resilience (22, 23). The total score ranges from 0 to 100, with higher scores indicating greater resilience (22, 23). Studies revealed the internal consistency for the CD-RISC scale as 0.89 (22) and 0.92 (24), respectively. Based on quartile deviation, resilience is scored as ≤ 50 representing the poor mental resilience and ≥ 51 representing the good mental resilience in this study.

The Perceived Stress Scale-10 (PSS-10) was used to measure the degree to which the participants identify events as stressful during the past month (25), with a good internal consistency of 0.78–0.91 (25–27). The total score ranges from 0 to 40, with higher scores indicating higher perceived stress (25).

### Statistical Analyses

All statistical analyses were performed with SPSS 22.0 software (IBM Corp, Armonk, NY, USA). Statistical significance was set as a *p*-value < 0.05 (two-tailed). Pearson's test and chi-square test were used to determine the distribution of categorical variables, and Mann–Whitney *U*-test was used for continuous variables. Univariate and multivariate analyses were performed to examine the correlates of depression using the logistic regression models.

## Results

### Sample Characteristics and Prevalence of Depression

In this study, 1,124 Chinese teachers were invited to participate in the survey. Twenty-eight participants were excluded as they completed the questionnaire in <120 s (*n* = 18) and were not 20–65 years old (*n* = 10). Finally, 1,096 teachers were enrolled with a median (range) age of 41 (20–65) years. Of them, 624 (56.9%) suffered from depression with a PHQ-9 score of >4, and 177 (16.1%) suffered from moderate to severe depression with a PHQ-9 score of >9. In addition, 117 (10.7%) teachers had poor mental resilience and 1,053 (96.1%) teachers had high perceived stress with a PSS-10 score of >13. Table 1 showed the socio-demographic and clinical characteristics between depression and no depression groups. We found that depression was more common among teachers who were of <41 years old (53.2 vs. 61.3%, *p* = 0.007), participated in epidemic prevention and control (52.7 vs. 59.1%, *p* = 0.042), with deterioration of parent-child relationship (56.1 vs. 71.9%, *p* = 0.019), thought distant teaching have bad effects (50.4 vs. 59.0%, *p* = 0.001), thought prolonged school closure have bad effects (49.2 vs. 60.3%, *p* = 0.014), had increased working intensity or time (52.5% vs. 60.3, *p* = 0.010; 52.9 vs. 60.7%, *p* = 0.009, respectively), had sleep duration/day of <6 h (69.6 vs. 54.4%, *p* < 0.001), had physical exercise duration/day of <30 min (64.1 vs. 48.7%, *p* < 0.001), spent less time with family (55.1 vs. 71.6%, *p* < 0.001), not concerned about COVID-19 (47.7 vs. 58.9%, *p* = 0.004), had poor mental resilience (53.3 vs. 87.2%, *p* < 0.001), and had higher PSS-10 score (16.3 vs. 39.1% vs. 67.0%, *p* < 0.001).

**Table 1:** Demographic characteristics of the study sample (*n* = 1,096).

**Variables**	**Total, *n* (%)**	**Depression**		***p*-value**
		**No, *n* (%)**	**Yes, *n* (%)**	
Gender				0.357
Female	871 (79.5)	369 (42.4)	502 (57.6)	
Male	225 (20.5)	103 (45.8)	122 (54.2)	
Age, year				0.007^*^
<41	511 (46.6)	198 (38.7)	313 (61.3)	
≥41	585 (53.4)	274 (46.8)	361 (53.2)	
Marital status				0.979
Unmarried	135 (12.3)	58 (43.0)	77 (57.0)	
Married	961 (87.7)	414 (43.1)	547 (56.9)	
Education background				0.974
Undergraduate or below	797 (72.7)	343 (43.0)	454 (57.0)	
Master or above	299 (27.3)	129 (43.1)	170 (56.9)	
Professional title				0.467
Senior	308 (28.1)	138(44.8)	170 (55.2)	
Intermediate or below	788 (71.9)	334 (42.4)	454 (57.6)	
School type				0.305
College school	262 (23.9)	120 (45.8)	142 (54.2)	
Senior high school or below	834 (76.2)	352 (42.2)	482 (57.8)	
Participating in epidemic prevention and control				0.042^*^
No	372 (33.9)	176 (47.3)	196 (52.7)	
Yes	724 (66.1)	296 (40.9)	428 (59.1)	
Being a parent				0.880
No	179 (16.3)	78 (43.6)	101 (56.4)	
Yes	917 (83.7)	394 (43.0)	523 (57.0)	
Deterioration of parent-child relationship				0.019^*^
No	1039 (94.8)	456 (43.9)	583 (56.1)	
Yes	57 (5.2)	16 (28.1)	41 (71.9)	
Teaching graduating class				0.568
No	900 (82.1)	384 (42.7)	516 (57.3)	
Yes	196 (17.9)	88 (44.9)	108 (55.1)	
Participating in distant teaching				0.240
No	183 (16.7)	86 (47.0)	97 (53.0)	
Yes	913 (83.3)	386 (42.3)	527 (57.7)	
Bad effect caused by distant teaching				0.001^*^
No	331 (30.2)	168 (50.8)	163 (49.2)	
Yes	765 (69.8)	301 (39.7)	461 (60.3)	
Bad effect caused by prolonged school closure				0.014^*^
No	262 (23.9)	130 (49.6)	132 (50.4)	
Yes	834 (76.1)	342 (41.0)	492 (59.0)	
Increased working intensity				0.010^*^
No	474 (43.2)	225 (47.5)	249 (52.5)	
Yes	622 (56.8)	247 (39.7)	375 (60.3)	
Increased working time				0.009^*^
No	524 (47.8)	247 (47.1)	277 (52.9)	
Yes	572 (52.2)	225 (39.3)	347 (60.7)	
Sleep duration/day, h				<0.001^*^
≥6	915 (83.5)	417 (45.6)	498 (54.4)	
<6	181 (16.5)	55 (30.4)	126 (69.6)	
Physical exercise duration/day, min				<0.001^*^
≥30	509 (46.4)	261 (51.3)	248 (48.7)	
<30	587 (53.6)	211 (35.9)	376 (64.1)	
Spending less time with family				<0.001^*^
No	983 (89.7)	441 (44.9)	543 (55.1)	
Yes	113 (10.3)	31 (27.4)	81 (71.6)	
Concerned about COVID-19				0.004^*^
No	901 (82.2)	370 (41.1)	529 (58.9)	
Yes	195 (17.8)	100 (52.3)	95 (47.7)	
Poor mental resilience				<0.001^*^
No	979 (89.3)	457 (46.7)	522 (53.3)	
Yes	117 (10.7)	15 (12.8)	102 (87.2)	
Perceived stress scale-10				<0.001^*^
0–13	43 (3.9)	36 (83.7)	7 (16.3)	
14–19	317 (28.9)	193 (60.9)	124 (39.1)	
20–40	736 (67.2)	243 (33.0)	493 (67.0)	

* *Statistically significant*.

### Characteristics Associated With Depression

Univariate logistic regression analysis in this study showed that participants who participated in epidemic prevention and control [odds ratio (OR) =1.298, 95% CI: 1.009–670, *p* = 0.042], suffered from deterioration of parent-child relationship (OR = 2.004, 95% CI: 1.110–3.618, *p* = 0.021), thought distant teaching have bad effect (OR = 1.563, 95% CI: 1.206–2.026, *p* = 0.001), thought prolonged school closure have bad effect (OR = 1.417, 95% CI: 1.072–1.872, *p* = 0.014), had increased working intensity or time (OR = 1.372, 95% CI: 1.078–1.747, *p* = 0.010; OR = 1.375, 95% CI: 1.082–1.748, *p* = 0.009, respectively), had sleep duration/day of <6 h (OR = 1.918, 95% CI: 1.362–1.748, *p* < 0.001), had physical exercise duration/day of <30 min (OR = 1.875, 95% CI: 1.472–2.389, *p* < 0.001), spent less time with family (OR = 2.152, 95% CI: 1.397–3.315, *p* = 0.001), had poor mental resilience (OR = 5.953, 95% CI: 3.413–10.38, *p* < 0.001), or had higher PSS-10 score (OR = 7.278, 95% CI: 3.209–16.51, *p* < 0.001) had higher odds of suffering from depression (Table 2). Those who aged ≥41 years (OR = 0.718, 95% CI: 0.564–0.914, *p* = 0.007), or were concerned about COVID-19 (OR = 0.635, 95% CI: 0.466–0.867, *p* = 0.004) were less likely to develop depression (Table 2). In the multivariate logistic regression model, participants aged ≥41 years (OR = 0.752, 95% CI:0.578–0.979, *p* = 0.034), participating in epidemic prevention and control (OR = 1.413, 95% CI:1.070–1.867, *p* = 0.015), thinking prolonged school closure have bad effect (OR = 1.385, 95% CI:1.017–1.885, p = 0.038), sleep duration/day of <6 h (OR = 1.814, 95% CI:1.240–2.655, *p* < 0.001), physical exercise duration/day of <30 min (OR = 1.619, 95% CI:1.247–2.103, *p* < 0.001), spending less time with family (OR = 1.729, 95% CI: 1.063–2.655, p = 0.002), concerned about COVID-19 (OR = 0.609, 95% CI:0.434–0.856, *p* = 0.004), poor mental resilience (OR = 6.570, 95% CI:3.533–12.22, *p* < 0.001) and high PSS-10 score (OR = 9.058, 95% CI:3.817–21.50, *p* < 0.001) represented the independent factors for suffering from depression (Table 2).

**Table 2:** Univariate and multivariate logistical regression analyses of correlates of depression.

**Variables**	**Univariate analyses**		**Multivariate analyses**	
	**OR (95%CI)**	***p*-value**	**OR (95%CI)**	***p*-value**
Age, ≥41 vs. <41	0.718 (0.564–0.914)	0.007^*^	0.752 (0.578–0.979)	0.034^*^
Participating in epidemic prevention and control, yes vs. no	1.298 (1.009–1.670)	0.042^*^	1.413 (1.070–1.867)	0.015^*^
Deterioration of parent-child relationship, yes vs. no	2.004 (1.110–3.618)	0.021^*^	1.064 (0.546–2.076)	0.854
Bad effect caused by distant teaching, yes vs. no	1.563 (1.206–2.026)	0.001^*^	1.104 (0.804–1.515)	0.541
Bad effect caused by prolonged school closure, yes vs. no	1.417 (1.072–1.872)	0.014^*^	1.385 (1.017–1.885)	0.038^*^
Increased working intensity, yes vs. no	1.372 (1.078–1.747)	0.010^*^	0.950 (0.669–1.349)	0.776
Increased working time, yes vs. no	1.375 (1.082–1.748)	0.009^*^	0.981 (0.690–1.395)	0.917
Sleep duration/day, <6 h vs. ≥6 h	1.918 (1.362–2.702)	<0.001^*^	1.814 (1.240–2.655)	0.002^*^
Physical exercise duration/day, <30 min vs. ≥30 min	1.875 (1.472–2.389)	<0.001^*^	1.619 (1.247–2.103)	<0.001^*^
Spending less time with family, yes vs. no	2.152 (1.397–3.315)	0.001^*^	1.739 (1.063–2.844)	0.027^*^
Concerned about COVID-19, yes vs. no	0.635 (0.466–0.867)	0.004^*^	0.609 (0.434–0.856)	0.004^*^
Poor mental resilience, yes vs. no	5.953 (3.413–10.38)	<0.001^*^	6.570 (3.533–12.22)	<0.001^*^
Perceived stress scale-10, 14–40 vs. 0–13	7.278 (3.209–16.51)	<0.001^*^	9.058 (3.817–21.50)	<0.001^*^

* *Statistically significant*.

## Discussion

Teaching was proved to be one of the most stressful jobs, which made teachers vulnerable to depression (7, 9). The outbreak of COVID-19 had a great negative impact on daily life and mental health among teachers, resulting in increased incidence of depression. Chinese is the largest ethnic group with one-fifth of the world population and has more teachers and students than other countries. In addition, higher levels of teachers' depressive symptoms were associated with poorer student psychological distress and well-being (15). Thus, early prevention, detection and management are essential to protect teachers from depressive disorder and improve their mental health. In the present online cross-sectional survey, we found that 56.9% of teachers suffered from depression during the outbreak of COVID-19 and 16.1% suffered from moderate to severe depression, which required follow-up diagnosis and treatment (19). In this study, the depressive symptoms were significantly related to age, participating in epidemic prevention and control, parent-child relationship, opinions toward distant teaching and prolonged school closure, increased working intensity and time, sleep duration/day, physical exercise duration, spending time with family, attitude toward COVID-19, mental resilience and stress. This result maybe highly informative for stakeholders to develop intervention efforts for high-risk groups.

A study of 1,210 respondents from 194 cities in China indicated that the prevalence of moderate to severe depression in the general population was 16.5% (28), which was similar to 16.1% in our study. However, as for the overall prevalence, a cross-sectional study of 1,006 Japanese teachers indicated that the prevalence of depression was 20.1% (29). Another research reported that 49.7% (21,797/43,845) Mexican teachers suffered from depression (30). We found a higher depression prevalence of 56.9%, which means that the COVID-19 outbreak is an important risk factor of depression for teachers. The emergence of COVID-19 has parallels with the outbreak of severe acute respiratory syndrome, which killed thousands infected patients in China. As an unpleasant experience, the outbreak of COVID-19 led to mass quarantine, fears of infection, stress, anger, boredom, frustration, lack of family and finance, all of which are related to increased incidence of depression (18, 31, 32). Since the COVID-19 outbreak was no longer confined to China, the problems associated with home confinement and school closure also became concerned in other affected countries.

To prevent the spread of COVID-19, the Chinese government ordered a nationwide school closure, and public activities were discouraged. Millions of teachers were confined to their homes, resulting in decreased outdoor activities and visits to others. During the COVID-19 outbreak, home confinement and prolonged school closure might have a significant negative impact on teachers' mental health, although these measures and efforts were highly commendable and necessary. Our results also indicated that less sleep duration and physical exercise were obviously associated with higher incidence of depression, which was in line with previous studies. Physical exercise was proved to be a promising antidepressant treatment (33). And three times of light to moderate intensive exercise a week for 6 to 12 weeks could bring alleviate depressive symptoms (34). The relationship between depression and short sleep duration was reported to be bidirectional (34). Additionally, accumulating evidences suggested that short sleep duration might be a prospective predictor of depression (35).

Our results indicated that those teachers who were younger, participated in epidemic prevention and control, had deterioration in parent-child relationship, held negative thoughts of distant teaching/prolonged school closure, spent less time with family or had increased workload had higher risk of depression. These teachers faced enormous pressure due to high workload, low income, fears of infection, uncertainty about how long they will last, low familiarity with remote education and lack of family (36), all of which were source of stress, which reduces the overall physical and mental well-being of teachers and leads to unpleasant emotions, such as depression (7). This may have led to the findings.

In the present survey, teachers concerned more about the outbreak of COVID-19 had lower risk of depression. Partly because clear communication and regular and accurate updates about COVID-19 could improve relevant knowledge of the disease and reduce the sense of fear and uncertainty (16). In addition, the National Health Commission of China released the national guideline of psychological crisis intervention for COVID-19, which could help teachers have a better understand COVID-19. Future studies, focused on those counterintuitive results, are needed.

Our results found that 96.1% teachers had high perceived stress, which was in somewhat in agreement with the findings of previous studies that teaching was one of the most stressful jobs (9). In this study, 10.7% teachers had poor mental resilience. Resilience may be believed to be a measure of stress coping ability and, could be an important target of treatment in depression (22). Resilience could embody the personal qualities that help one to thrive in adversity (22). This might be the reason why those teachers with high perceived stress and poor mental resilience have high risk of depression in this study.

In this study, several limitations should be noted. First of all, this is an online study, those with no access to the Internet could not join. Secondly, some data of important factors associated with depression, such as social support and physical health, were not available due to logistical reasons. Thirdly, due to the nature of cross-sectional study, causality between variables could not be assessed. Meanwhile, this study used a snowball sampling which might influence the sample's representation and lead to sampling bias. Last but not least, this study was unable to do the comparison of the difference before and after the outbreak due to the lacking of follow-up data. Thus, a well-designed study of the prevalence of depression is necessary to complement epidemiological studies and provide more conclusive evidence regarding the risk and correlative factors of depression among teachers during the COVID-19 outbreak.

## Conclusion

Depression was common among teachers during the COVID-19 outbreak. Age, participating in epidemic prevention and control, opinions toward distant teaching and prolonged school closure, sleep duration/day, physical exercise duration, spending time with family, attitude toward COVID-19, mental resilience and stress represented the independent factors for suffering from depression.

## Data Availability Statement

The raw data supporting the conclusions of this article will be made available by the authors, without undue reservation.

## Ethics Statement

The studies involving human participants were reviewed and approved by the Medical Ethical Committee in Beijing Anding Hospital of the Capital Medical University, China. The patients/participants provided their written informed consent to participate in this study.

## Author Contributions

XC and JL designed the study. JZ and XY wrote the protocol, analyzed the data, and wrote the first draft of manuscript with input from HY. HH and YL commented on the protocol. All authors contributed to the article and approved the submitted version.

## Conflict of Interest

The authors declare that the research was conducted in the absence of any commercial or financial relationships that could be construed as a potential conflict of interest.
